# A Case of Polysarcia, Reported for the Buffalo Medical and Surgical Journal

**Published:** 1861-11

**Authors:** Ira D. Hopkins

**Affiliations:** Physician and Surgeon City Hospital, Utica, N. Y.


*A Case of Polysarcia, reported for the Buffalo Medical and Surgical Journal, by Ira D. Hopkins, M. D. Physician and Surgeon City Hospital, Utica, N. Y.*

Richard Holmes, colored, a cook, aged 41 years, was admitted into the Utica City Hospital on the 14th of August, 1861, suffering from polysarcia, of which he died September 3d, 1861. He was 5½ feet high, weighed about 350 pounds ; and measured in circumference 4 feet 10 inches, other portions of his body were in equal proportions. For 14 years he clothed himself in female apparel.

On post-mortem examination, assisted by Drs. Coventry and Thomas, found two inches of fat covering the chest, from the clavicular to the abdominal extremity of the sternum; from the abdominal extremity of the sternum to the umbilicus, two and a half inches of fat; from the umbilicus to the pubis, three inches of fat; his breast was as large as any female breasts that I ever saw ; the skin of all the unexposed parts of the body, was hard and carrugated ; upon opening the thorax, found the lungs sound, but very small ; the left lung weighed $12\frac{1}{2}$ oz ; the heart was large, weighed 24 oz. but was perfectly sound. Opening the abdomen, found all in a healthy condition, but some of the organs were larger than usual ; the liver weighed five pounds ; the kidneys were about equal in size, and one of them weighed 12 oz. ; the spleen was very small, did not weigh it ; the penis and testacles were very small, not larger than they are usually found in boys of seven or eight years of age ; the scrotum was $1\frac{1}{2}$ inches thick.

